# Preparation and In Vitro Evaluation of Protective Effects of Quercetin‐Loaded Solid Lipid Nanoparticles on Human Hair Against UV‐B Radiation

**DOI:** 10.1111/jocd.16566

**Published:** 2024-09-09

**Authors:** Anayatollah Salimi, Behzad Sharif Makhmalzadeh, Mandana Salahshoori

**Affiliations:** ^1^ Nanotechnology Research Center Ahvaz Jundishapur University of Medical Sciences Ahvaz IR Iran; ^2^ Department of Pharmaceutics, Faculty of Pharmacy Ahvaz Jundishapur University of Medical Sciences Ahvaz IR Iran

**Keywords:** hair, quercetin, SLN, UV‐B radiation

## Abstract

**Background:**

The aim of this study was to investigate the protective effect of quercetin loaded on solid lipid nanoparticles (SLN) in protecting human hair from ultraviolet‐B (UV‐B) light in vitro.

**Methods:**

In this study, solvent‐emulsified diffusion method was used to fabricate nanoparticle formulations and then particle size, loading, and drug release tests were performed from different formulations. Variables include oily part proportion, liquid to solid oil part ratio, and surfactant to lipid ratio. The optimal formulation was prepared by examining the eight formulations and optimizing them. Six groups of hair with different treatments were exposed to UV light for 600 h and the changes were investigated by examining four factors: RMS (root mean square average, the microscopic profile peaks and valleys), peak to valley roughness, the amount of chemical changes by Fourier transform infrared spectroscopy (FTIR), and the amount of protein loss.

**Results:**

The selected formulation had a suitable particle size, loading percent, and release rate for penetration to hair. Quercetin‐loaded SLN controlled RMS factor, peak to valley roughness, and reduced chemical changes and protein loss compared to other treatments.

**Conclusion:**

The optimize formulation showed positive effects in protecting the hair strands from UV‐B radiation.

## 1 Introduction

Flavonoids, as strong antioxidants and anti‐radicals, are polyphenolic compounds mainly found in plants, whose antioxidant power depends on the position of the hydroxyl group in the flavonoid skeleton. Quercetin is a flavonol from the flavonoid group of polyphenols with antioxidant and anti‐inflammatory properties found in most fruits, vegetables, leaves, and seeds [1], which is expected to have a sunscreen effect. Direct exposure of the hair to sunlight and ultraviolet (UV) rays, especially UV‐B, can seriously damage hair follicles, particularly the cuticle layer. To protect hair from sun damage, suitable hair care products must be designed [2]. The intensity of the effect of sunlight on hair depends on various factors, such as wavelength, radiation length, hair color, hair structure, including hair thickness, hair composition, and so forth [3]. The function of hair is to protect from the sun's UV rays, repel insects, and ensure beauty. Although hair protects the body from UV rays, it becomes brittle and even discolored; thus, we must protect our hair from the sun's rays. Solar radiation includes UV (wavelength: 400–280 nm), visible light or VIS (wavelength: 700–400 nm), and infrared or IR (wavelength: 2800–750 nm). Most of the energy of the sun's rays comes from the range of UV rays (wavelength: 200–200 nm), which is divided into three categories based on wavelength: UV‐A (wavelength: 400–315 nm), UV‐B (wavelength: 315–280 nm), and UV‐C (wavelength: 280–100 nm) [4]. Since the damaging effect of UV light (especially UV‐B light) on hair has been proven, designing hair care products against sunlight can be highly beneficial. Richena et al. investigated the effect of mercury lamp radiation on hair. They irradiated healthy hair for 500 h (irradiated for 10 h and kept in the dark for 14 h). The observed morphological changes included an increase in the height of the edge of the scales, the appearance of a bump on the cuticle surface, and the enlargement of holes on the hair surface. The protein oxidation rate increased, while the lipid oxidation rate did not change significantly [3].

There is an average of 100 000–150 000 strands of hair on each adult's head (100 000 for black hair and 150 000 for blonde hair). Hair diameter is between 50 and 80 μm, which varies in different races. Hair life varies depending on the location of hair growth. Hair grows at a rate of 0.1–0.4 mm/day. There are two types of hair on the human body: (1) vellus hair that is colorless, soft, small hair covering most of the body, and (2) terminal hair or pigmented hair that is thicker and found on the head, beard, armpits, and pubis. The different parts of the hair shaft from outside to inside are the cuticle, cortex, and medulla, each of which has a different role where hair care products exert their effect [5].

The cuticle is divided into three parts, the layers of which are from the outside to the inside of the epicuticle, A‐layer, exocuticle, and endocuticle. The epicuticle is the outermost layer of the cuticle rich in cysteine (about 12% w/w). The cysteine groups of the epicuticle layer are attached to a lipid layer (18‐methyleicosanoic acid or 18‐MEA) and form a proteolipid membrane that coats the cuticle. The A‐layer and exocuticle layers are hydrophobic and have a high cysteine content (A‐layer 30% w/w and exocuticle 15% w/w). The endocuticle is the innermost layer where the cysteine content is 3% w/w [3]. The cortex layer forms the main hair space. The mechanical properties of hair are reliant on the cortex layer. The cortex forms the major part of the hair and contains melanin, which determines hair strength, flexibility, color, and texture. The medulla is the innermost layer of the hair shaft, which is only present in thick hairs. Compared to other parts of the hair, it is rich in fat and citrulline but low in cysteine. The hair growth cycle consists of three distinct phases called anagen (the active phase of hair growth), catagen (the intermediate phase of hair growth), and telogen (the resting phase of the hair or hair loss) [6].

Solid lipid nanoparticles (SLNs) have been introduced as carriers for active cosmetic ingredients, pharmaceutical drugs and herbal ingredients. It has been shown that they act as active carriers for sunscreens due to their particulate character. SLNs are sub‐micron colloidal carriers ranging from 50 to 1000 nm, which are composed of physiological lipid, dispersed in water or in aqueous surfactant solution. SLN offer unique properties such as small size, large surface area, high drug loading and the interaction of phases at the interface. SLNs are categorized as classical SLN, polymer‐lipid hybrid nanoparticles (PLN), nanostructured lipid carriers (NLC), and lipid‐drug conjugate (LDC). SLNs are considered as one of the most appropriate carriers for the delivery of lipophilic ingredients (such as quercetin) as they ensure high drug encapsulation and controlled release properties [7, 15].

Flavonoids refer to highly effective antioxidant compounds. Quercetin is a flavonoid with a special role in sunscreen products and low cell membrane permeability. The formulation of this material in classical SLN can increase permeability. In this study, the protective effect of quercetin‐containing SLN against UV‐B light was investigated.

## 2 Materials and Methods

### 2.1 Materials

Our material for this study consisted of Quercetin from Solarbio (China), Tween 80, lecithin, oleic acid, propylene glycol, methanol, benzyl alcohol, di‐sodium hydrogen phosphate dihydrate, sodium di‐hydrogen dihydrate from Merck (Germany), Transcutol P (diethylene glycol monoethyl ether) and Comprito l888 ATO (glyceryl dibehenate) from Gattefossé's (France).

### 2.2 Methods

#### 2.2.1 SLN Production Method

We prepared our SLN with the solvent‐emulsified diffusion method as described further, benzyl alcohol and water was mixed in the ratio of 1:1 for 10 min in 47° and then kept for 20 min to be saturated from each other. Then the lipid part (includes oleic acid and Compritol 888 ATO) and half of the surfactant (a mixture contains Tween 80 and lecithin in the ratio of 1:1) were solved in the water‐saturated organic part (in 47°) and then quercetin and the surfactant residue along with propylene glycol and transcutol were added to 8 mL of benzyl‐saturated water compound in the same temperature. At the next step, we added the lipid part to the water potion and mixed it for 15 min by homogenizer with a velocity of 2000 rpm to form an emulsion. Twenty milliliters of water at 4° was added to the emulsion while homogenizing and then homogenized for another 30 min. At last, they were mixed for 10 min at 4° in an ultrasonic bath (sonicator) to form the SLN [7, 8].

Consumed lipids: Compritol 888 ATO and oleic acid.

Consumed Surfactant: a mixture contains Tween 80 and lecithin in the ratio of 1:1.

Consumed co‐solvent: propylene glycol.

Cosolvent: Transcutol P.

In this study, a factorial design with three independent variables at two levels was used to prepare the eight formulations. Our variables in this production method include lipid percent (%Lipid), liquid to solid oil part ratio (L oil/S oil), and surfactant to lipid ratio (Sur/L).

The ratios of the variables used in each formulation are presented in Table 1. The optimized formulation composition was determined with Minitab 17 software to find the level of independent variables that would obtain a minimum value of particle size and the maximum amount of drug loading. The value of particle size and drug loading are critical parameters that determine quercetin concentration and penetration into hair as the main site for protection against UV‐B.

**TABLE 1:** The amount of variables used, the particle size, polydispersity index, amount of loading, and particle size after 3 months in each formulation (mean ± SD, *n* = 3).

Formulation number	%Lipid	L. oil/S. oil	Sur/Lip	Particle size (nm)	Polydispersity index	%Loading particle size (after 3 months)	%Loading particle size (after 3 months)
1	90%	0.1	0.05	46.72 ± 2.85	0.385 ± 0.01	54.93 ± 0.1	47 ± 1.2
2	90%	0.1	0.025	39.04 ± 1.61	0.332 ± 0.03	56.96 ± 2.13	39.5 ± 0.7
3	90%	0.05	0.05	41.80 ± 2.34	0.39 ± 0.01	50.86 ± 0.66	42.05 ± 0.5
4	90%	0.05	0.025	40.04 ± 1.06	0.375 ± 0.02	55.25 ± 0.14	40.6 ± 0.8
5	50%	0.05	0.025	34.53 ± 0.71	0.315 ± 0.01	40.57 ± 0.33	34.8 ± 0.45
6	50%	0.1	0.05	44.7 ± 3.10	0.404 ± 0.05	39.12 ± 0.24	45.01 ± 1.1
7	50%	0.05	0.025	29.67 ± 0.86	0.342 ± 0.03	40.01 ± 0.14	29.92 ± 0.7
8	50%	0.1	0.05	35.52 ± 1.59	0.365 ± 0.06	38.99 ± 0.4	36.1 ± 0.9

#### 2.2.2 SLN Features Survey

##### 2.2.2.1 SLN Particle Size Survey

Particle size was measured by SCATTER SCOPE 1 QUIDIX (South Korea) based on photon correlation spectroscopy at 25°C with a wide measurable size range (1–7000 nm). Three samples of each formulation were taken and measured three times [9].

##### 2.2.2.2 SLN Loading Rate Survey

We used direct and indirect methods to measure the loading level of the drug. After formulations preparation, an initial suspension containing SLN was poured into high round centrifuge cells, placed in fridges of centrifuges at 4° at a velocity of 20 000 rpm for 30 min. Then dispersed particles were washed with a certain amount of solvent and centrifuged again. We mixed the superficial portions in each step and used them in wavelength of 382 to determine the unloaded quercetin in the indirect method. The difference between the entire added drugs into formulation from the free drug (superficial part) is defined as the loaded drug. In the direct method, we took a defined amount of sediment and solve in a certain amount of methanol to achieve the amount of loaded drug [10, 11]. The used equation of standard curve is *Y* = 9.2557*x* − 0.0004 and regression co‐efficient (*R* ^2^) = 0.9975.

##### 2.2.2.3 Drug Release Rate Survey in the Selected Formulation

This survey was performed in vitro by using diffusion‐standing cells. A certain amount of sediment is achieved by centrifuge diluted with a certain amount of solvent. An equivalent volume of each formulation was taken and placed on membranes. Acetate cellulose membrane with a cutoff of 12 kDa fixed on the cells and drug loading levels were measured at hours of 0.5, 1, 2, 4, 6, 7, 8, 24, and, 48 [12, 13].

#### 2.2.3 Survey of UV‐B Effect on Samples

##### 2.2.3.1 Samples Preparation and Grouping

Dark brown hairs of a female volunteer were washed with a milliliter of a water solution of 0.2% sodium lauryl sulfate and drained for 30 s. This process was repeated twice. Then the hairs were dried at room temperature and were stored in a plastic bag until use.

Groups included the following:

Group 1: treated hairs with optimized 1% quercetin formulation

Group 2: treated hairs with an optimized blank formulation (quercetin free)

Group 3: treated hairs with quercetin solution in 1% concentration solvent (phosphate buffer pH = 7.4 and methanol in the ratio 1:4)

Group 4: treated hairs with MY commercial sunscreen with SPF = 50 (positive control)

Group 5: treated hairs with solvent alone (phosphate buffer pH = 7.4 and methanol in the ratio 1:4)

Group 6: hairs without any treatment

0.15 g of hairs was washed and dried as considered above, placed for 5 min in the targeted formulation, then drained for 2 min, dried afterward and exposed to UV‐B at the end. A sample of hairs was exposed to UV‐B without any contact to formulations.

##### 2.2.3.2 UV‐B Radiation Protocol

Hairs will be exposed to radiation of UV‐B with an intensity of 1.6 J/cm for 10 h a day and left for 14 h in darkness. This process will be repeated for 20, 30, 40, 50, and 60 days. The radiation intensity is controlled with a radiometer. Hairs will be kept in an aluminum‐covered container to improve radiation navigation.

##### 2.2.3.3 Survey of Peak to Valley Roughness Between Coticule Scales and RMS Roughness Factor With Atomic Force Microscopy (AFM) Method

Hair strand images will be prepared in non‐contact mode with a scanning probe microscope. These images will be taken before and after radiation at room temperature. Then roughness factors image including peak to valley roughness between coticule scales and RMS roughness factor (according to the following equation) is achieved by the processing software [3].

$$\mathrm{RMS}=\sqrt{\frac{\sum {\left(Z_{n}-Z_{\mathrm{ave}}\right)}^{2}}{N}}$$

where *z*_ave_ is the average of the *z* value within a specific area, *z*_*n*_ is the *z* value for a given point within this area and *N* is the number of measured points.

##### 2.2.3.4 Survey of Chemical Conversion of Samples With Fourier‐Transform Infrared Spectroscopy (FTIR)

We surveyed six strands of hair with 4 cm^−1^ resolution separately before and after UV‐B exposition. Their spectrums were obtained and were interpreted for their chemical changes according to their intensity variations and spectrums locations [3].

##### 2.2.3.5 Survey of Hairs Protein Loss

0.15 g of hairs was placed in a 10‐mL Erlenmeyer flask and 2.5 mL of water was added to it and kept for 30 min at room temperature in a sonicator to investigate radiation effect on hair proteins. Then the superficial solution was separated and analyzed for total protein with a UV–Vis spectrophotometer according to the Bradford protein assay. We used bovine serum albumin to construct our standard solution [14, 15].

## 3 Results

### 3.1 Results of the Properties of the Formulations of the Produced SLN

#### 3.1.1 Particle Size Results of SLN

The particle size results of the formulations containing the drug are presented in Table 1.

The highest particle size is for formulation number 1 (46.72 ± 2.85 nm) and the lowest particle size is related to formulation number 7 (29.67 ± 0.86).

##### 3.1.1.1 Investigation of the Effect of Independent Variable on the Particle Size of Quercetin‐Loaded SLN

Simultaneous multiple regression analysis was used to investigate the effect of independent variables on particle size. The following equation shows this relationship.

PZ = 15.9 + 0.151% lipid + 105 l. oil/s. oil + 127 sur/lip.

Analysis of this equation shows that between the variables % Lipid (*p* = 0/004) and l. oil/s. oil (*p* = 0/009) have a significant and direct relationship with particle size.

#### 3.1.2 Results of SLN Loading Percent

The loading results of quercetin‐containing lipid nanoparticles are presented in Table 1.

The highest loading rate is related to formulation number 2 (56.96% ±2.13). The lowest load is related to formulation number 8 (38.99% ± 0.4).

##### 3.1.2.1 Investigation of the Effect of Independent Variable on Quercetin Loading in the Formulation of SLN

The relationship between the independent variables and the loading percentage is expressed by the following equation:

%Loading=22.7+0.367%lipid+13.3l.oil/s.oil−61.7sur/lip.

Analysis of this relationship shows that the relationship between %Lipid and Sur/Lip variables with loading percent is significant. The percentage of lipid is directly related to the amount of loading and Sur/Lip is inversely related to the amount of loading.

### 3.2 Selecting the Optimal Formulation and Examining Its Properties

By examining the characteristics of the formulations in Table 1, the optimize formulation was selected. The characteristics of the optimal formulation are presented in Table 2.

**TABLE 2:** The amount of variables, particle size, loading, and release of the drug in the optimize formulation (mean ± SD, *n* = 3).

%Lipid	L. oil/S. oil	Sur/lip	Particle size	%Loading	%*R*_*1*_	%*R*_*2*_
72.09%	0.079	0.025	36.02 ± 1.21	50.67 ± 1/12	15.93 ± 1.71	91.02 ± 0.40

### 3.3 Results of the Effect of UV‐B Light on Hair

#### 3.3.1 Peak to Valley Roughness

**FIGURE 1:**
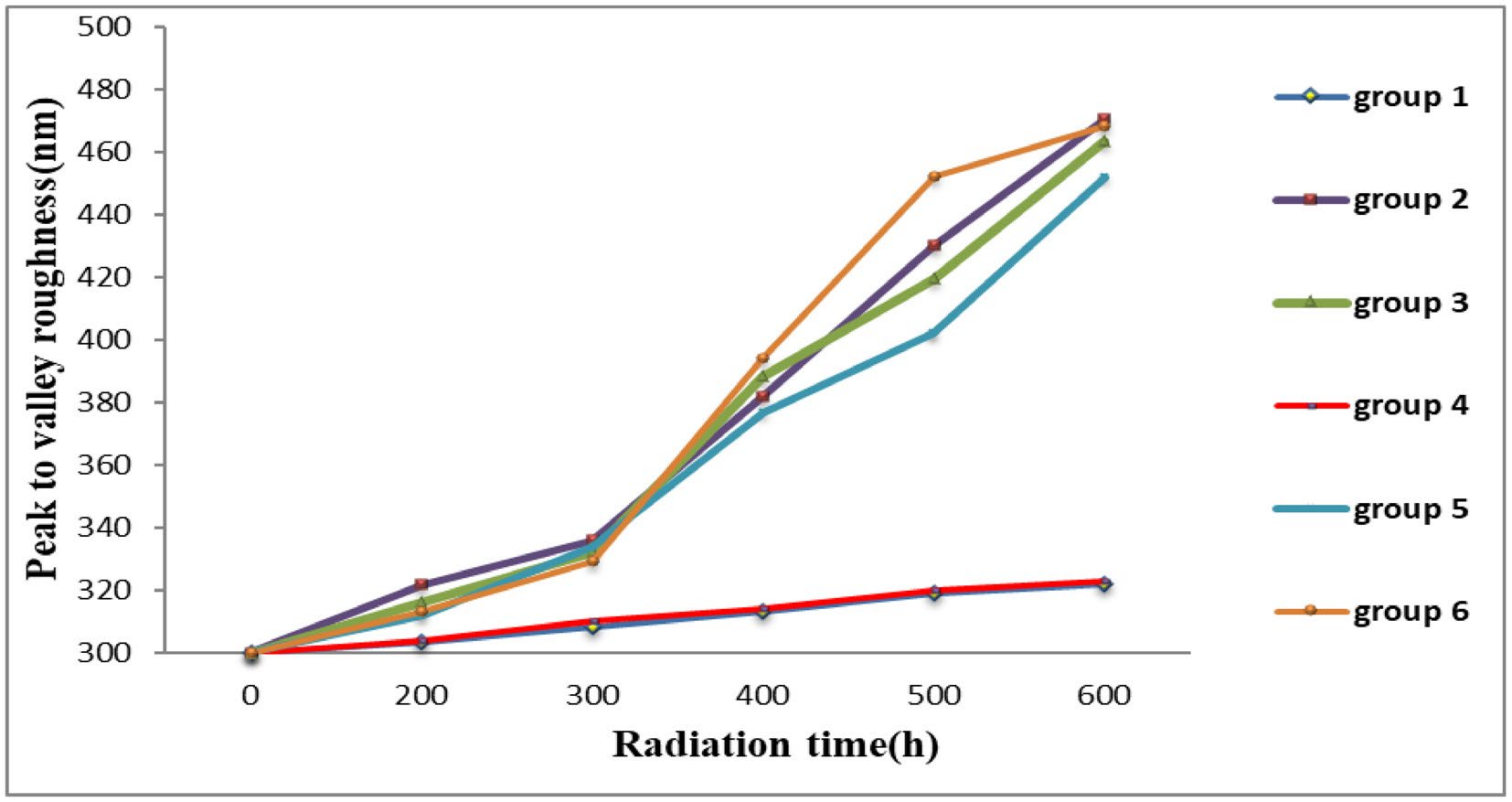
Peak to valley roughness changes in six groups according to radiation time.

**FIGURE 2:**
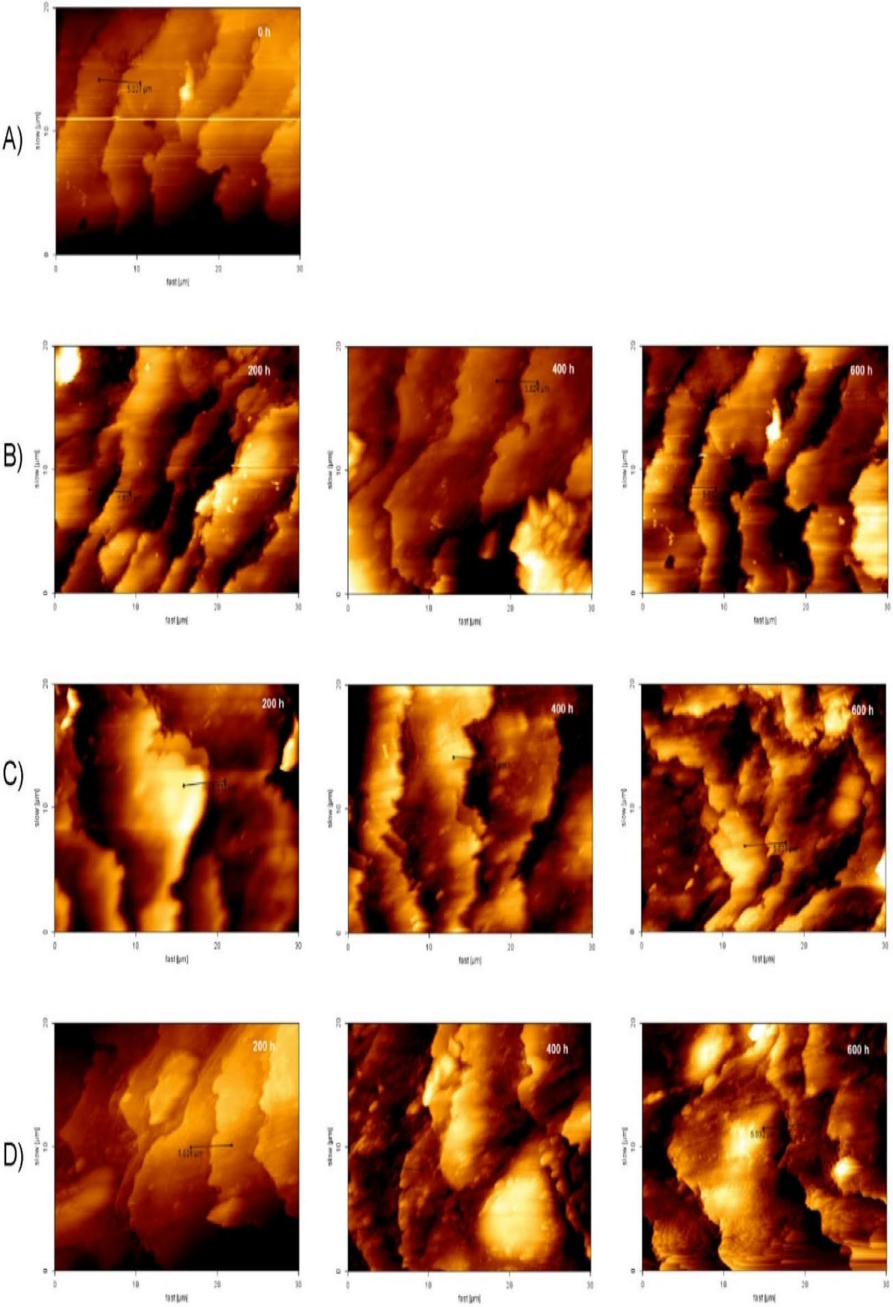
AFM results of (A) untreated and unirradiated hair; (B) negative control group; (C) group receiving the optimized formulation and (D) positive control group (the duration of radiation is specified in the images).

The results of peak to valley roughness changes and RMS roughness of hair surface with AFM imaging technique in the six groups of hairs are presented in Figures 1 and 2, respectively. Peak to valley was seen in all groups after 600 h of UV radiation. In Groups 2, 3, 5, and 6, the increase in the height of the edge of the scales was statistically significant but in Groups 1 and 4, the increase in the height of the scales was not significant (*p* = 0.21 and 0.4, respectively). As a result, UV light could not cause hair destruction in these two groups. Comparing the results of 600 h radiation time of each group with the time of 600 h radiation time of negative control groups shows that only Groups 1 and 4 are statistically significantly different from the negative control group. A comparison of Groups 1 and 4 shows that these two groups are not statistically significant. As a result, the two groups are equally effective in protecting human hair from UV light.

#### 3.3.2 RMS Roughness

**FIGURE 3:**
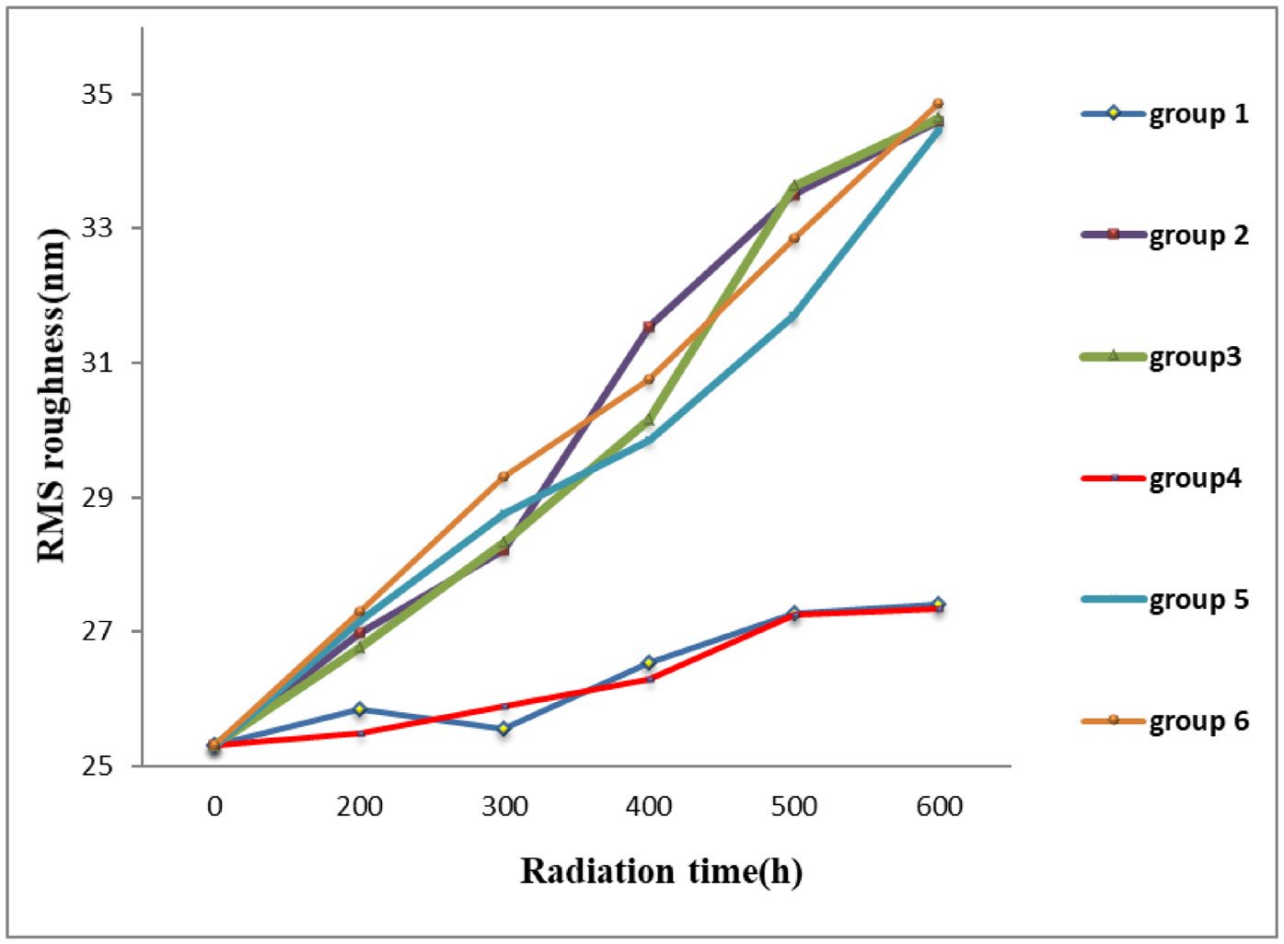
RMS roughness changes in six groups according to radiation time.

The graph of RMS roughness changes in the six experimental groups is presented in Figure 3. The process of change in RMS roughness is like the peak to valley roughness. In all Groups except 1 and 4, the changes during radiation are statistically significant. UV radiation was able to significantly increase the roughness in Groups 2, 3, 5, and 6. But the severity of changes in Groups 1 and 4 is not statistically significant (*p* = 0.001 and 0.02, respectively). Optimal formulation and positive control groups have been able to reduce the severity of the destructive effects of UV light on hair. There is no statistically significant difference between Groups 1 and 4 and both groups have almost the same effectiveness. Considering that the drug alone and the selected formulation without the drug each separately did not prevent the destruction of hair follicles, so it can be concluded that the presence of SLN formulation was effective for the penetration of the drug into the hair follicles.

#### 3.3.3 FTIR

One of the methods to study chemical changes in hair due to UV radiation is the use of FTIR technique. The following peaks can be seen in a healthy undamaged hair:

Peak wavelengths of 1041 cm^−1^ due to oxidation of disulfide bonds in hair keratin, 1076 cm^−1^ due to C–C vibrations, 1237 cm^−1^ related to amide III bands, 1518 cm^−1^ due to N–H tensile vibrations with part of C–H, 1633 cm^−1^ caused by C–O vibrations amide I and 3287 cm^−1^ due to O–H tensile vibrations [3].

**FIGURE 4:**
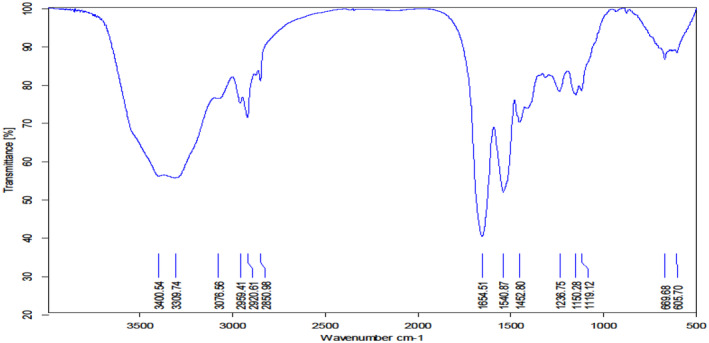
FTIR spectra of negative control group on Day 0.

All of the mentioned peaks can be seen with a slight displacement in Figure 4.

According to a study by Richena et al., on the FTIR spectrum of healthy human hair, two peaks, 1041 cm^−1^, which is a measure of cysteine oxidation, and peak 2926 cm^−1^, which indicates the amount of lipids [3]. In the present study, the same peaks were used to investigate the destructive effect of UV light. In the present study, the peak of cysteine oxidation occurred in the range of 1078–1081 cm^−1^. The intensity of this peak in negative control samples increased significantly from Days 0 to 60, indicating that UV light was able to increase the rate of cysteine oxidation. In the positive control samples after 20 and 60 days and the optimized formulation after 20 and 60 days, the severity of this peak was significantly reduced. In blank formulation, the reduction in peak intensity is less than in Groups 1 and 4. Therefore, it seems that blank formulation also had a somewhat protective effect. The results show that the effectiveness of the positive control group is higher than the optimal formulation group.

The results show that none of the formulations caused a significant change compared to the negative control group in peak 2926 cm^−1^. Therefore, the selected formulation has a suitable protective effect mainly on the S=O group in the disulfide bond and has prevented its oxidation.

#### 3.3.4 The Rate of Protein Loss

**FIGURE 5:**
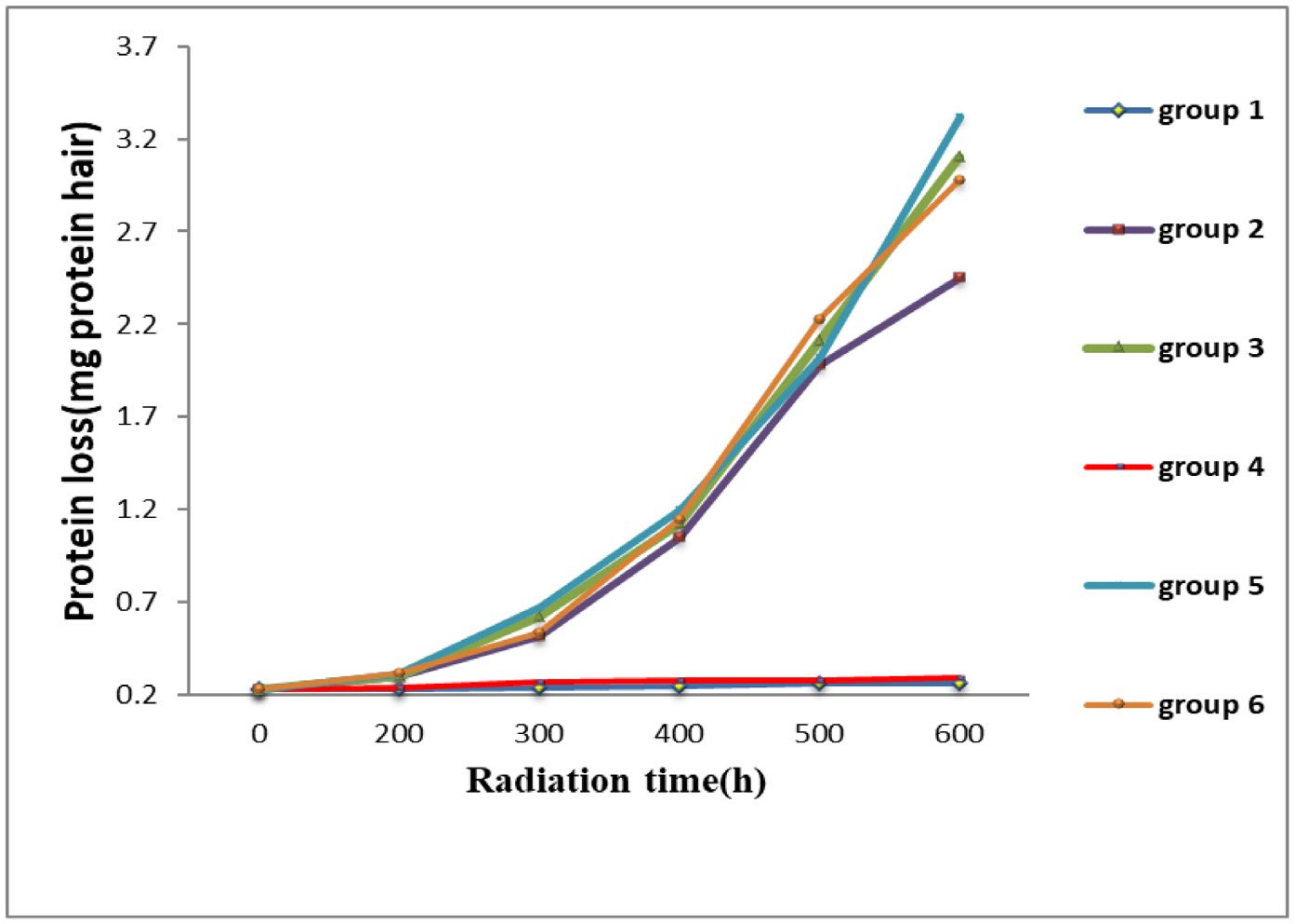
The rate of protein loss in six groups according to radiation time.

To calculate the amount of protein in each hair sample, a standard diagram of bovine serum albumin in water was used. The results of protein loss are shown in Figure 5.

The amount of protein lost in Groups 1 and 4 was significantly reduced compared to the negative control group. As a result, the two groups of positive control and optimal formulation had a good protective effect on the hair but other groups were not significantly different from the negative control group.

## 4 Discussion

Quercetin has low solubility in water and low permeability of membranes, so in order to improve the solubility of the drug and increase the permeability of the drug through the hair, we used the SLN carriers. For determining the optimized formulation, eight formulations were prepared with different lipid percentage, liquid oil to solid oil ratio, and surfactant to lipid ratio. The results shows that between the variables % Lipid (*p* = 0/004) and L. oil/S. oil (*p* = 0/009) have a significant and direct relationship with particle size. Also, shows that the relationship between %Lipid and Sur/Lip variables with loading percentage is significant. Lipid percentage is directly related to the amount of loading percentage and Sur/Lip ratio is inversely related to the amount of loading percent. Thus, the addition of a stabilizer to the formulation is extremely important as it decreases surface tension and the surface energy of the system while imparting a repulsion force between particles. Therefore, the suitable choice of stabilizer type and concentration is crucial to ensure the fabrication of highly stable SLNs. According to previous literature, different types of stabilizers having different molecular weight and charge have been successfully utilized to produce stable SLNs [16]. Tween 80 and lecithin in our formulations in stabilized SLNs showed remarkably lower particle size. A lower particle size of Tween 80‐stabilized SLNs was also previously reported. Tween 80 has an HLB value of 15. A smaller particle size obtained in the case of Tween 80‐stabilized nanoparticles could be attributed to its lower HLB value [17].

Smaller particle size and higher loading percent are more suitable for the selected formulation. So the selected formulation was prepared with 72.09% lipid, 0.079 ratio of liquid oil to solid oil, and 0.025 ratio of surfactant to lipid. The loading percent of the selected formulation was 50.67 ± 1.12 mg/mL and also particle size = 36.02 ± 1.21 nm; which indicates the suitable loading and particle size of the selected formulation. In our research, %Lipid with loading percent is significant which may be explained by its highly lipophilic nature of quercetin [16].

To determine the effectiveness of the selected formulation, the results of peak to valley roughness, RMS, FTIR (shows chemical changes), and the amount of hair protein loss in six sample groups were compared.

Previously, Richena and Rezende studied the effect of photodamage on the outermost cuticle layer of human hair and reported that with UV radiation with an intensity of 1.6 J cm^−2^ on human hair, after 500 h, a significant increase in peak to valley roughness and RMS was created [3]. The results of the negative control group showed that hair damage increased over time and the peak to valley roughness increased significantly, especially after 300 h of radiation. While both the optimized formulation and the positive control formulation have prevented the increase of peak to valley roughness and its graph has not changed significantly even after 600 h of UV radiation compared to zero time. The protective effect of the optimized formulation was significantly higher than the positive control group from 300 h of radiation onward. It was also found that the placebo formulation (Group 2) had no protective effect. Therefore, the observed protective effect is related to optimized formulation of quercetin (Group 1); and the SLN as a carrier has increased the durability of quercetin on hair.

The trend of changes in RMS was also similar to peak to valley roughness. In Groups 2, 3, and 5, similar to the negative control group, this parameter shows a visible increase after 600 h of UV‐B radiation. This is while the optimized formulation containing quercetin has been able to effectively prevent the increase of this parameter similar to the positive control group. From the fact that in Groups 2 and 3, quercetin and the formulation separately could not provide effective protection, it can be concluded that the formulation caused this protective effect by increased the durability of quercetin on hair. The obtained results confirm those by Richena and Rezende [3].

The results of the FTIR spectrum of hair presented that UV‐B radiation destroyed hair structure, especially structures such as S=O group, amide III, C–H bond of protein and lipid parts, and amide I. All the structural changes were created especially from Day 20 to Day 60 of UV‐B radiation. UV‐B has been able to induce the oxidation of the amino acid cysteine, which is effective in changing hair color. In this study, both the optimized formulation (Group 1) and the positive control formulation (Group 4) have been able to prevent these structural changes from occurring, and the superiority of the optimized formulation over the positive control formulation was significant. Also, the placebo formulation has protective effects and can prevent the oxidation of cysteine to some extent. Therefore, the carrier and quercetin have strengthened each other's effect in protecting hair. In the previous studies by Richena et al. the results of a study on the FTIR spectrum of hair showed that with 500 h of UV radiation on hair, the oxidation of cysteine has increased, which leads to chemical changes in hair protein structure. But the lipid structures of hair did not change significantly which was agreed with the results obtained in our research [3]. In our study, after the 60th day of UV‐B radiation, the examination of the peak related to S=O bond has shown an increase in the oxidation of this group in the negative control group, and from the fact that such a change is observed in Groups 3 and 5, it can be concluded that UV‐B radiation has increased the oxidation of this bond in the untreated samples and caused chemical changes in them.

The results of several studies present that UV radiation on hair with a destructive effect on proteins increases their solubility with increasing radiation time [18, 19]. Also, the results of another study proved that the effect of the UV‐B spectrum is more significant than other sunlight spectrums in increasing the solubility of proteins [14]. In the protein loss test, it was found that in Groups 2, 3, 5, and 6, with the passage of radiation time, the amount of protein loss increased significantly but in Groups 1 (optimized formulation) and 4 (positive control) no protein loss were observed after 600 h of radiation. So, these two groups have shown an acceptable protective effect to reduce the amount of hair protein loss caused by UV‐B radiation.

## 5 Conclusion

Based on the obtained results, quercetin can be introduced with the dose proposed in this study as a molecule that protects hair against UV‐B radiation, as well as the SLN formulation, both by increasing the durability of quercetin on hair and by having protective effects can be introduced as a suitable carrier for quercetin in the design of hair care formulations. The SLNs containing quercetin had better effects compared to the positive control and it was able to effectively prevent damage caused by UV‐B radiation in hair. Since the quercetin solution and blank solution (free of quercetin) could not provide good protection to hair in the tests, it was concluded that SLN is a suitable formulation for protection of hair against UV‐B radiation by quercetin molecule.

## Conflicts of Interest

The authors declare no conflicts of interest.

## Data Availability

Research data are not shared.
